# Contributions to the Treatment of Croup

**Published:** 1858-08

**Authors:** 

**Affiliations:** Director of the Children’s Hospital of Mariahilf, Vienna


*Contributions to the Treatment of Croup.* By Dr Luzinsky, Director of the Children's Hospital of Mariahilf, Vienna.

Translated from Journal fur Kinder Krankheiten, by Dr. Maleech.

There are few diseases which the physician is called upon to treat that are more prevalent than angina membranosa, or which engages his attention to a greater degree; for the reason that there are few diseases more terrible and dangerous to the infantile sufferer than this—seizing the child in the bloom of health, and too often terminating rapidly its young career of life. As yet, science has done but little to ameliorate the symptoms and lessen the danger attending this formidable disease; with its earnest endeavors and the torrents of literature called forth bythe fatality of the complaint, she has, as yet, gained but a trifle, but, on the other hand, we may safely say that our confusion in regard to the treatment and pathology of this malady daily increases. A careful examination of this disease, in the forms usually presented, has led me to the belief that error in its diagnosis is not uncommon, two other forms of disease being often mistaken for that of true croup. I refer to laryngeal catarrh and laryngeal spasm, separate or combined, occurring in the same person. Strict systematizers are prone to regard these latter and somewhat related affections of the larynx, (so often running into each other) as but stages of angina membranosa, which appears to me most erroneous, and diagnosis on those premises cannot terminate other than with disadvantage in a practical point of view.

Careful search for the causes of our present confusion touching the disease in question will develope the fact that it is not the different phases which the complaint takes on that enhances our troubles, for those phases are sufficiently definite, but it lies rather with the various authors who have attempted its solution, and who have not given that due regard to its pathological anatomy which it deserves, or who do not manifest that close circumspection in their works so necessary to the clear demonstration of an abstruse subject One author teaches that croup is simply a spasmodic affection of the larynx. He will place the same title on laryngismus stridulus or asthma laryngeum; he will find in his autopsy false membrane similar to that of croup, (though not the real false membrane of this disease,) and assert, in cases where those products are met with, that it resulted from spasm alone. These are the doctrines taught by Haherkopff. Others, again, see croup in every cough, while others still, equally learned, do not attribute to these conditions the slightest importance, but wait for further symptoms to justify the diagnosis of croup.

From that inattention to pathological anatomy which is too common, we have a cause for many of the discrepancies of authors, and while they have boasted of brilliant success in the treatment of croup, they could, in truth, speak only of sorrowful results.

In the Austrian journals (Jan. 1855, p. 6-7,) I have published the results of my studies on angina membranosa, its reality, appearance and treatment, and will here quote but a small portion of that paper for the purpose of connecting my subsequent investigations:

"That angina membranosa depends upon an inflamed condition of the mucous membrane of the air passages, pathological anatomy most clearly teaches. Redness of the larynx alone is most common, th ough it often extends to the trachea and bronchia; there is also a thickened condition of the membrane, a plastic, slimy mucous, or, in its place, larger or smaller quantities of pseudomembrane. Besides these conditions, there is often found (as I have repeatedly observed) similar appearances on the gums, the inner surface of the lips on pharynx, tonsils, aœsphagus, the heart, on the stomach and intestine, in the female genital organs, and even in the bladder; they are, at times, met with on the skin behind the nose, in the axilla, and on abraded surfaces or sores. Angina membranosa is often attended with more or less croupose pneumonia. These circumstances furnish decisive proof of the existence of a peculiar crasis from whence those croupose symptoms and pseudo-membranes arise, and teach us that we cannot consider croup a local disease, but simply the local expression of a general diathesis. It is undeniable that a spasmodic condition of the larynx exists coincident with the inflammatory state of the organ; it occurs, as a sequel to the inflammatory stage, and is therefore a secondary symptom, though not the less important, for it constitutes one of the violent symptoms of the disease, and demands particular attention in treatment."

In proof of our views upon these points, above quoted, I will state that those authors who have furnished the pathological conditions present in their cases of fatal termination, have shown the trifling material changes found in the larynx are wholly insufficient to account for the violence of the symptoms during life and the ensuing death. In the *Journal fur Kinder Krankheiten*, (1856, p. 9-10,) Schlautmann endeavored to establish the doctrine that the symptoms of this disease were due to paralysis of the laryngeal muscles; but, however elegant and scientific his explanations, they are not confirmed by experience, for, on one hand the phenomena of periodicity in the supervention of the cough and dyspnœa, and on the other hand the favorable effects of opiates militate against him.

Without entering minutely into the well knwon symptomology of angina membranosa, I will speak of its prominent features: Croup is characterized by a sharply hoarse voice, and a dry, harsh cough, which is so well marked as to have obtained for it the name of "croupal sound;" it is of such peculiarity that after having heard it once it is difficult to forget. A sound most similar to this is that of the barking of a young dog. These two symptoms—hoarseness and cough—are accompanied with difficult respiration and more or less fever. These symptoms, with which croup always begins, have not as yet received their due estimation by many physicians. An otherwise highly esteemed gentlemen in science has expressed himself in the following manner: "It is a peculiarity of some children to cough from every cold, in this hoarse, barking manner." Mauthner asserts that cough, and even a simple bronchial catarrh, assumes in children with short necks, the croupy sound, and that it is met with in those cases suffering from worms. These purely theoretical assertions have not been once confirmed at our wards during fifteen years' practice on many thousands of children. They are usually unsustained in practice as they are wanting in rational physiology. I would not deny that hoarseness, cough and fever attend laryngeal catarrh. These symptoms often become troublesome, but they will usually disappear easily under simple treatment; those of croup, however, constantly advance, and are attended with dyspnœa, which is not the case with the former complaint. We can thus see why authors make so little difference between catarrhal and the true pseudo-membranous disease. Weber has ably defended this ground in the Deutschen Klinik, 1854, p. 23-4. The peculiar "croupy sound" is the first and also the most important symptom indicating the inception of the disease; it continues to its termination. When it is heard we should regard it as a warning to be mindful of danger, and if the parent and physician would bestow upon it that attention which it merits there would be but few infants comparatively, who would fall a sacrifice to the malady.

In the treatment of this disease careful attention should be given to the four following points:

*First* —To change the Crasis present.

*Second*—To prevent localization of inflammation in the larynx.

*Third*—To cut short the spasms of the larynx.

*Fourth*—To throw off the pseudo-membranes.

There are but few at the present day who will not admit that croup is dependent on a peculiar condition of the blood—a crasis whereby it becomes in itself more plastic, and exhibits a greater tendency to form false membrane on mucous surface; therefore, our first efforts should be to break in upon this morbid tendency. Such remedies, then, as induce a more fluid condition of the blood are plainly indicated. Those which have proved most useful in our wards to fulfil this indication, and at the same time unobjectionable from any peculiarity of condition induced in other parts of the system through their action, (such remedies as antimony, mercurials, sulph cupi. etc.,) are the carbonates of potassa and soda: they possess the power of dissolving albumen and fibrinous products, (recent researches in physiology have proved this fact,) and have long been used in diseased conditions of a similar nature with much advantage. I have for many years used these salts in croup with the most happy results, and in 1855, in the Austrian journals, gave publicity to the details of their action.— They received the sanction of the French, and Mareschal, in Paris, in 1856, published a memoir on their salutary effects even in the advanced stages of the disease. Jort, Valentin and Mareschal, in the *Un. Med.*, 1856-7, have confirmed the successful use of these remedies when other treatment had failed. It is proper to state that Valentin used the cautery in some of his cases at the same time that the alkaline salts were being exhibited. The dose of these remedies which I am in the habit of prescribing, is from one and a half to two drachms of either salt in twenty-four hours, according to the age of the patient; this should be dissolved in two ounces of distilled water, and after its solution add one ounce of simple syrup. The remedy is continued till the cough becomes loose, and as soon as this occurs a plastic, puss-like slime is thrown off.

[*Pacific Med & Surgical Journal.*]

